# Draft Genome Sequence of the Nonstarter Bacteriocin-Producing Strain *Enterococcus mundtii* CRL35

**DOI:** 10.1128/genomeA.00444-14

**Published:** 2014-05-22

**Authors:** Julieta Bonacina, Lucila Saavedra, Nadia E. Suárez, Fernando Sesma

**Affiliations:** Laboratorio de Genética y Biología Molecular, Centro de Referencia para Lactobacilos (CERELA-CONICET), San Miguel de Tucumán, Tucumán, Argentina

## Abstract

*Enterococcus mundtii* CRL35 is a bacteriocinogenic strain isolated from an artisanal cheese of northwestern Argentina. Here we report its draft genome sequence, consisting of 82 contigs. *In silico* genomic analysis of biotechnological properties was performed to determine the potential of this microorganism to be used in a food model system.

## GENOME ANNOUNCEMENT

The microorganisms of the genus *Enterococcus* are present in many fermented foods such as cheeses, meats, and olives, where they play a fundamental role in the development of the characteristic sensory profile and extension of shelf life (1, 2).

*Enterococcus mundtii* CRL35 is a bacteriocinogenic nonstarter lactic acid bacterium (LAB) strain isolated from an artisanal cheese of northwestern Argentina that produces enterocin CRL35, a bacteriocin active against *Listeria monocytogenes* (3–8).

The *E. mundtii* CRL35 genome was sequenced using a whole-genome shotgun (WGS) strategy (286,971 total reads of ~111.5 Mb, with 34.83× genome coverage) with a 454 GS Titanium pyrosequencer at the Instituto de Agrobiotecnología de Rosario (INDEAR), Argentina. The resulting nucleotide sequences were *de novo* assembled into 82 contigs, with a mean contig size of 34,972 bp, using the 454 Newbler 2.6 assembler (454 Life Sciences, Branford, CT). Only 60 contigs were longer than 1,000 bp in size. The *N*_50_ contig length was 108,098 bp, the largest contig assembled was 326,582 bp, and the shortest was 101 bp. The draft genome sequence consists of 2,867,684 bp with an estimated genome size of 3.2 Mb and a mean GC content of 37.98% ± 4.74%.

Genomic analysis was done using the RAST annotation server (9), Blast algorithms, ISGA (10), BACTIBASE (11), and BAGEL3 (12). Results obtained with RAST showed that there are 305 subsystems denoted in the chromosome, which represent only 40% of the sequences assigned. A total of 2,778 coding sequences (CDS) and 58 structural RNAs (55 tRNAs) were predicted.

An *in sílico* genomic screening of biotechnological properties demonstrated that the *E. mundtii* CRL35 genome contains genes involved in lactose utilization and uptake (*lacR*, *lacC*, *lacE*, *lacF*, *lacG*, and *lacZ*), oligopeptide transport systems (*oppA*, *oppB*, *oppC*, *oppD*, and *oppF*), aminopeptidase S (*pepS* with Leu, Val, Phe, and Tyr preference), isoaspartyl dipeptidase (Asp-X-specific dipeptidase), aminopeptidase C (*pepC*), proline dipeptidase, methionine aminopeptidase, and aminopeptidase YpdF. A set of genes related to lipase and esterase activities, such as those for GDSL-like lipase/acylhydrolase, phospholipase D, tributyrin esterase, glycerophosphoryl diester phosphodiesterase, acyl-acyl carrier protein (ACP) thioesterase, and phospholipase/carboxylesterase, was also localized. An NADP-specific glutamate dehydrogenase gene related to the production of flavor compounds (13) was located in contig 3, and no genes responsible for nitrate and nitrite reductase activities were found.

This study confirmed the presence of an enterocin CRL35 biosynthetic cluster, and BAGEL3 software analysis demonstrates that this would be the only bacteriocin cluster present in this strain.

Many reports suggest that enterococci might act as opportunistic pathogens (14), so an *in silico* analysis of virulence determinants was performed (3). In this sense, the following genes, an aggregation substance gene (*agg*), an enterococcal surface adhesion gene (*ace*), an enterococcal surface protein gene (*esp*), a gelatinase gene (*gelE*), the cytolysin operon (*cylA*, *-B*, *-M*, or *-L*), and *fsrB* for the *fsr* quorum-sensing system, were evaluated (15, 16). None of the tested virulence genes were identified.

Functional studies are being conducted to determine the use of this microorganism in the food industry.

### Nucleotide sequence accession numbers.

This whole-genome shotgun project has been deposited at DDBJ/EMBL/GenBank under the accession number JDFT00000000. The version described in this paper is version JDFT01000000.
